# An efficient signature aggregation scheme with antenna hardware implementation for VANETs

**DOI:** 10.1038/s41598-025-04035-y

**Published:** 2025-07-02

**Authors:** A. Priyadharshini, Arun Sekar Rajasekaran, Kalyan Sundar Kola, Arunkumar Balakrishnan, Radha Mohan Pattanayak

**Affiliations:** 1 Department of Computer Science and Engineering, Coimbatore Institute of Technology, Coimbatore, Tamil Nadu India; 2 Department of Electronics and Communication Engineering, SR University, Warangal, Telangana 506371 India; 3 Lab-STICC,Department of Electronique, Universite de Bretagne Occidentale, Brest, 29238 France; 4 Department of Computer Science and Engineering, Brainware University, Kolkata, West Bengal 700125 India; 5 Department of Information Technology, Manipal Institute of Technology Bengaluru, Manipal Academy of Higher Education, Manipal, India; 6 Department of Computer Science and Engineering (SCOPE), VIT-AP University, Amaravati, 522237 India

**Keywords:** Authentication, Integrity, Miniaturization, Printed antenna, Privacy, Signature, Engineering, Mathematics and computing

## Abstract

Vehicular ad-hoc networks (VANETs) plays a major role in enhancing road safety and traffic management. However, they suffer from security and scalability issues due to dynamic network conditions. Conventional signature aggregation schemes have high computational and communication cost limiting real-time efficiency. To overcome this issue, an efficient mutual certificateless anonymous authentication with signature aggregation is presented in this work. Moreover, a compact printed antenna using concentric octagonal rings based on the ‘Golden ratio’ principle is designed in this work. This antenna is used for the transfer of location-based information (LBI) at a frequency of 5.90 GHz inside the Dedicated Short-Range Communications (DSRC) band (802.11p) between the On-board unit (OBU) of the vehicle user and the roadside unit (RSU). Further, formal and informal security investigation shows the resistance of the proposed protocol against well-known attacks. Moreover, the efficiency of the suggested protocol is analysed in terms of security attributes, computational and communication overhead using the Cygwin platform with respect to relevant works. Finally, the performance of the designed antenna for the transfer of LBI is validated based on return loss, impedance bandwidth, gain, cross-polarization discrimination, back-lobe radiation, and radiation efficiency using the Computer Simulation Technology (CST) microwave studio (v2019).

## Introduction

Vehicular Ad Hoc Network (VANET) is an advanced form of Mobile Ad Hoc Network (MANET) specifically designed to facilitate communication between vehicles (V2V) and between vehicles and infrastructure (V2I) in a dynamic, rapidly changing vehicular environment. It leverages the emerging technologies of wireless communication and the Internet of Things (IoT) to create a connected and intelligent transportation system. Vehicles equipped with VANET technology can form a self-configuring network without relying on a fixed infrastructure. This enables real-time data exchange and information sharing among nearby vehicles and roadside units, leading to enhanced road safety, improved traffic management, and more efficient transportation^1^.

The history of VANET can be traced back to the late 1990s and early 2000s, when researchers began exploring the concept of vehicle-to-vehicle communication to improve road safety and traffic management. Initial studies focused on using DSRC for communication between vehicles and infrastructure^2^. In 2002, the U.S. Federal Communications Commission (FCC) allocated a frequency band specifically for DSRC applications, further promoting VANET research and development. This allocation encouraged automotive manufacturers to invest in VANET-related technologies. In the following years, international standardization bodies like IEEE started developing VANET-related standards, including IEEE 802.11p, which further solidified VANET’s foundation.

One of the primary applications of VANET is to improve road safety. Vehicles within the network can share crucial location-based information (LBI), such as their speed, direction, and braking status, allowing others to anticipate potential collisions and take preventive actions. This real-time data exchange is particularly valuable for avoiding accidents and mitigating traffic congestion. Moreover, VANET facilitates smart traffic management and reduces traffic congestion. Traffic data collected from various vehicles can be used to optimize traffic flow, suggest alternative routes, and regulate traffic signals dynamically. This not only enhances the overall driving experience but also reduces fuel consumption and emissions. Moreover, VANET paves the way for the integration of connected and autonomous vehicles, making future transportation more intelligent and efficient.

To enable communication in VANET, various wireless communication technologies, including DSRC and Cellular Vehicle-to-Everything (C-V2X) technologies, are used. These technologies ensure low-latency, reliable, and secure data transmission within the network^3–5^. Although it is recognized that newer communication paradigms like C-V2X are rapidly replacing IEEE 802.11p-based DSRC technology, DSRC is still used in many real-world VANET deployments worldwide, especially to ensure backward compatibility. Furthermore, the suggested authentication and aggregation system can be easily modified to accommodate new V2X technologies without requiring significant adjustments, as it is primarily independent of the underlying physical layer. The contribution is therefore still applicable to both existing and developing automotive network infrastructures. In the context of VANET, a miniature antenna is a small antenna that is used to transmit or receive radio waves (5.9 GHz) between vehicles. Miniature antennas are often used in VANETs because they are small and lightweight, making them easy to install on the OBU of the vehicles or RSU^6^. Miniature antennas are typically used for DSRC, such as V2V and V2I communication. The choice of miniature antenna for a VANET application depends on a number of factors, such as the desired range of communication, the required data rate, and the available space on the vehicle. Moreover, in this work, a proposal has been made for the use of a compact printed antenna in the context of VANET applications, specifically designed to function inside the DSRC band (802.11p)^7^.

VANET is already proving to be useful in various scenarios and will continue to gain significance as technology advances. Some key situations in which VANET is and will be particularly beneficial include: Road Safety: VANET enhances road safety by enabling vehicles to share real-time information, such as location, speed, and braking status, with nearby vehicles. This data exchange helps prevent collisions and alerts drivers about potential hazards, significantly reducing the number of accidents. Traffic Management: VANET facilitates intelligent traffic management by collecting data from vehicles and using it to optimize traffic flow, suggest alternate routes, and dynamically adjust traffic signals. This leads to reduced congestion and more efficient transportation. Emergency Services: In the event of accidents or emergencies, VANET can enable faster response times by alerting emergency services and providing real-time information about the situation on the road. Cooperative Driving: VANET paves the way for cooperative driving, where vehicles can communicate and coordinate with each other, making driving safer and more efficient. Intersection Management: VANET can improve intersection management by allowing vehicles to communicate with traffic lights and other infrastructure, optimizing the traffic flow, and reducing wait times. Pedestrian Safety: VANET can also be used to enhance pedestrian safety by allowing vehicles to detect pedestrians and warn drivers when someone is crossing the road. Public Transportation: VANET can be employed in public transportation systems to improve fleet management, monitor bus schedules, and provide real-time updates to passengers. Smart City Integration: As cities become smarter and more connected, VANET will play a crucial role in integrating vehicles into the broader IoT infrastructure, enabling seamless communication and data exchange for various smart city applications.

As the technology continues to evolve and more vehicles become equipped with VANET capabilities, its usefulness will extend further, enabling a more connected, efficient, and safer transportation ecosystem^8^. However, VANET faces several challenges, such as ensuring data privacy and security, dealing with network scalability in densely populated urban areas, and handling high mobility and rapidly changing topologies. Although emerging paradigms like ORAN (Open RAN) and massive Machine Type Communications (mMTC) present future directions for vehicular communications, ensuring efficient authentication for current VANET infrastructures remains critically important for real-world deployments.

By lowering communication overhead and computational delays, the suggested aggregate signature approach with antenna hardware implementation meets the essential requirements of existing VANET systems and makes authentication procedures quicker, more scalable, and more effective. These enhancements are essential for enabling real-time vehicle communication in situations with high traffic, where conventional approaches frequently fail. Our method not only closes a significant gap in current VANET security frameworks but also paved way for real-world implementation in Intelligent Transportation Systems (ITS) by maximizing both hardware performance and cryptographic efficiency^9–12^.

The main contributions of this work are.

- To design a miniaturized printed antenna module with high gain and full bandwidth coverage to establish secure communication between RSU and vehicle users.
- To provide an anonymous certificateless mutual authentication between the vehicle user and RSU for efficient transfer of LBI between them.
- To preserve the integrity of LBIs, an aggregate signature validation scheme is presented.
- To conduct an exhaustive security and efficiency analysis to illustrate that the proposed protocol fulfills the security requirements for the VANET system.

The remainder of the manuscript is structured as follows: Sect. 2 reviews some of the relevant works, while Sect. 3 provides some necessary background data related to preliminaries and the VANET system model. Proposed work in terms of registration, antenna design, mutual authentication, signature generation, verification, and aggregate validation is described in Sect. 4, and the security model of the suggested schemes is shown in Sect. 5. Section 6 discusses the efficiency analysis of the VANET system in terms of computational and communication overhead. Moreover, practical implementation of the designed miniature antenna is also briefed in this section. Finally, Sect. 7 closes the work with a conclusion.

## Related works

Different researchers have addressed security and privacy. Some of the related works are as follows: The security assaults in VANETs are examined by Riya et al.^13^. They also underlined the significance of security procedures for protecting vehicular communications. They first introduced the concept of a conditional privacy-preserving authentication scheme. Later, Raya and Hubaux^14^ introduced the concept of public key infrastructure. The main drawback is the loading of a large number of certificates into the onboard unit of the vehicle. This increases the communication and storage overhead significantly. The security protocol can be constructed using two schemes, namely bilinear pairing and Elliptic curve cryptographic techniques.

Zhang et al.^15^ suggested an identity-based conditional privacy scheme based on a bilinear pairing scheme. Here, an identity-based signature is designed to preserve conditional privacy. Moreover, batch verification is used to enhance the verification efficiency. Further, key management plays a crucial role in security. Liu et al.^16^ suggested an efficient anonymous key agreement scheme. This scheme combines both bilinear pairing and trusted computing to safeguard against different security assaults. But the computational overhead of this scheme is very high. Further, the scheme can be easily traceable and vulnerable to internal attacks. To overcome this drawback, Bagga et al.^17^ suggested an authentication scheme in which the vehicles are authenticated only by RSU. The main drawback is the transfer of plain text messages. There is no encryption process, which makes messages accessible to everyone.

Huang et al.^18^ suggested a batch authentication key agreement scheme. This scheme authenticates a large number of vehicles using different session keys at the same time. Moreover, the scheme is based on the Elliptic curve cryptographic technique, which reduces the computational overhead drastically. Wang et al.^19^ suggested a key agreement protocol that is completely self-certified. Though batch authentication is achieved in this work, this scheme is based on bilinear pairing operations. This increases the computational overhead, which affects the performance of the system. Dua et al.^20^ suggested a two-level authentication key agreement protocol. A cluster head is introduced, which is responsible for the launch of session keys to the vehicles. However, the communication overhead of this scheme is high. Islam et al.^21^ suggested a password-oriented conditional privacy scheme for VANETs. The trusted authority should be online during the key update process. The new updated key can be computed as the update of the key is performed based on a simple encryption process. This may lead to a lack of security.

Liu et al.^22^ suggested a secure group key agreement scheme that relies on roadside units. The computational complexity and communication overhead of this scheme are high as it depends on bilinear pairing operations, which degrade the performance of the system. Ma et al.^23^ suggested an authentication scheme without the involvement of bilinear operations. However, this scheme suffers from an increase in storage cost.

Ming- Chung et al.^24^ proposed a multiple-strip-based printed monopole antenna for numerous wireless applications, including DSRC (IEEE 802.11p) systems. The antenna gives 3 dB gain and offers around 75% radiation efficiency, respectively. Fakharian et al.^25^ presented a monopole bat-shaped patch antenna for DSRC applications. The antenna gives a maximum gain of 1.8 dB and 82% radiation efficiency, respectively. Zhang et al.^26^ introduced a revolutionary mmWave antenna array design that use Concomitant Multifold Orthogonal Beamforming (CMOB) to enable efficient V2V and V2I communication. The concept provides high-gain, orthogonal beams without complex feeding networks by activating several cavity modes and incorporating slotted surfaces, which have been proven through simulation and testing. T. Limbasiya and D. Das^27^ introduced QueryCom, a lightweight and secure protocol for message exchange, data storage, and information retrieval in Vehicular Cloud Computing (VCC) environments. QueryCom uses SHA-512, AES-256, and an index-based search mechanism to assure data confidentiality, user anonymity, and efficient performance on resource-constrained devices. H. Zhang et al.^28^ presented a green communication strategy for terahertz networks that use high-gain directional antennas located outside from the service region to improve beam coverage in high-speed vehicular scenarios. The strategy enhances energy efficiency and cost-effectiveness by widening beam projection while eliminating complex phased arrays, as evidenced by performance analysis and simulations.

However, the suggested protocol achieves a signature aggregation scheme with low communication and computational cost. Moreover, the proposed printed antenna offers better gain and higher radiation efficiency compared to the reported literature, as mentioned above.

## Preliminaries

The preliminaries and system model of VANET are described in this section.

### Elliptic curve cryptography

A curve $\:{E}_{q}:\:{x}^{3}+\alpha\:x+\beta\:\:mod\:q$ over the finite field $\:{Z}_{q}$ such that $\:(\alpha\:,\beta\:)\in\:{Z}_{q}^{*}$ defines the ECC, given a large prime $\:q$. The numbers $\:\alpha\:$ and $\:\beta\:$ are chosen to satisfy $\:4{\alpha\:}^{3}+27{\beta\:}^{2}\:mod\:q\ne\:0$. The cyclic group $\:G$ is made up of the set of all points over the $\:{E}_{q}$. The point addition $\:X+Y$ yields another point $\:Z$if $\:X=\left({r}_{\alpha\:},{s}_{\alpha\:}\right)$and $\:Y=\left({r}_{\beta\:},{s}_{\beta\:}\right)$ are the two points of $\:{E}_{q}\left(\alpha\:,\beta\:\right)$. $\:Z=\left({r}_{\gamma\:,}{s}_{\gamma\:}\right)$, $\:{r}_{c}={\lambda\:}^{2}-{r}_{\alpha\:}-{r}_{\beta\:}\:mod\:q$ and $\:{s}_{\gamma\:}=\left(\lambda\:\left({r}_{\alpha\:}-{r}_{\gamma\:}\right)-{s}_{\alpha\:}\right)\:mod\:q$, where $\:\lambda\:$ can be computed respectively

$$\:\lambda\:=\left\{\begin{array}{c}\frac{{s}_{\beta\:}-{s}_{\alpha\:\:}}{{r}_{\beta\:}-{r}_{\alpha\:}}mod\:q\:if\:X\ne\:Y\\\:\frac{3{r}_{\alpha\:}^{2}+\alpha\:}{2{s}_{\alpha\:}}mod\:p\:if\:X=Y\end{array}\right.$$

Elliptic Curve Discrete Logarithm Problem (ECDLP): It is challenging to calculate $\:\beta\:\in\:{Z}_{q}^{\text{*}}$ given that the two random points on the elliptic curve $\:{E}_{q}$ are described by $\:X,Y\in\:{E}_{q}$ and $\:Y=\beta\:X$.

### System model

**Fig. 1:**
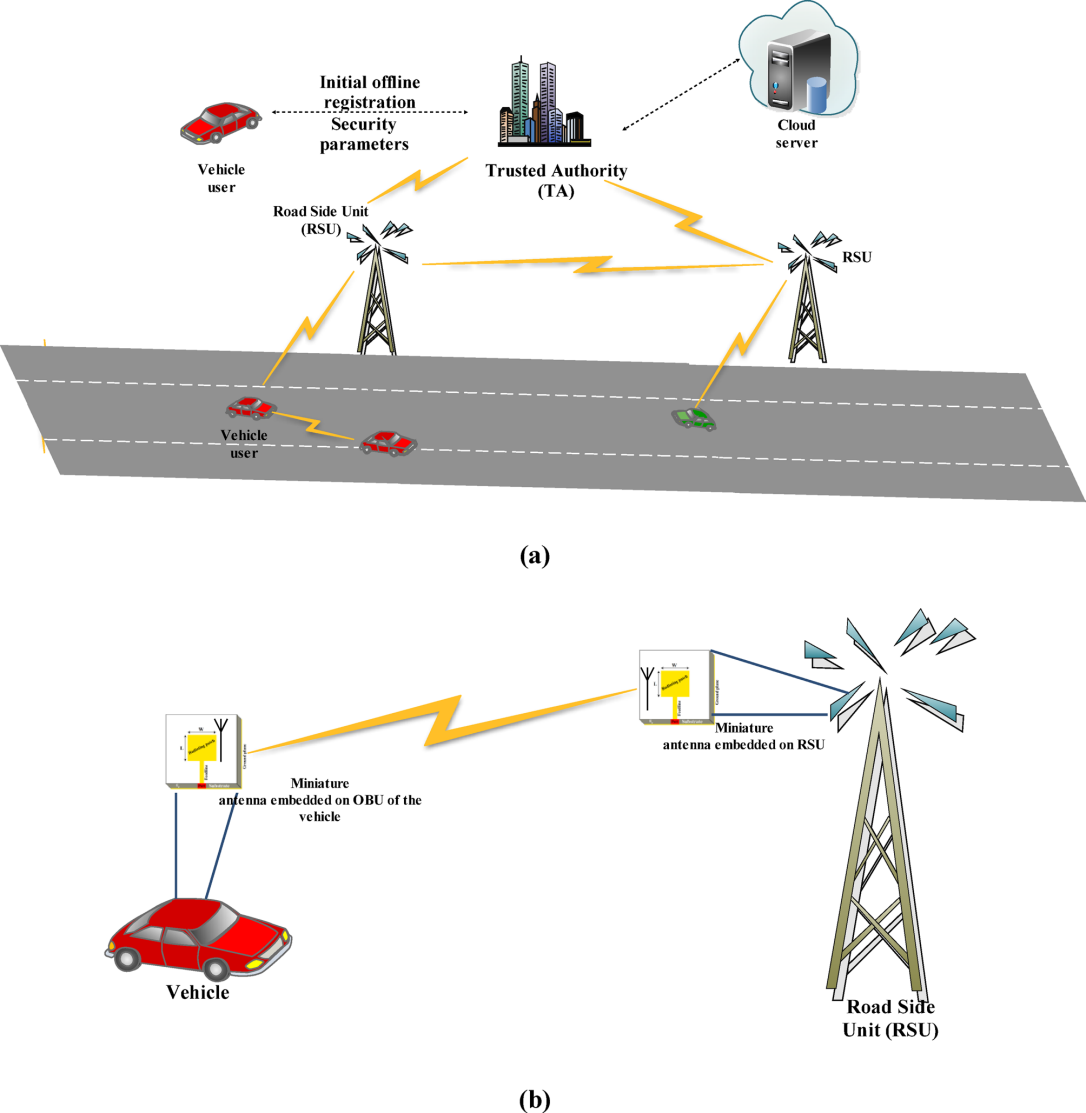
(**a**) VANET architecture, (**b**) Transfer of LBI with the help of miniature Antenna between OBU of vehicle and RSU.

The system model of the suggested scheme consists of three main entities: the vehicle user, the RSU, and the trusted authority (TA). The architecture of the VANET system is shown in Fig. 1a, followed by the transfer of LBI through the miniature antenna present in the OBU of the vehicle and the RSU shown in Fig. 1b as follows.

#### TA

TA is a fully trusted body and it cannot be compromised. It is responsible for establishing and managing trust relationships between $\:{V}_{i}$ and RSUs. It evaluates the trustworthiness of $\:{V}_{i}$ based on their behavior, communication history, and other factors. TA issues and manages digital certificates for $\:{V}_{i}$ and RSUs. These certificates are used to authenticate the identity of $\:{V}_{i}$ and ensure secure communication.Moreover, it manages the distribution and revocation of cryptographic keys used for secure communication between $\:{V}_{i}$ and RSUs. Further, TA monitors the behavior of $\:{V}_{i}$ in the network to detect and respond to malicious attacks.

#### Vehicle user$\:\left({V}_{i}\right)$

$\:{V}_{i}$ form the main entity in the system model of our suggested work. All the $\:{V}_{i}$ must be registered with the TA. Only registered and validated vehicle users are allowed to participate in the system. The $\:{V}_{i}$ use mobile sensors which are attached to the vehicles and they collect data from the RSU through the designed miniature antenna attached to the OBU. They can share this data in the DSRC range with other vehicles or the infrastructure with the help of this antenna to enhance situational awareness and improve safety. Further, $\:{V}_{i}$ play a role in maintaining the security and privacy of the VANET. Moreover, $\:{V}_{i}$ need to validate the received messages and forward reliable and accurate information to maintain the integrity of the network.

#### RSU

Initially, RSU should be registered with the TA. RSUs act as information hubs, disseminating essential safety and non-safety information to vehicles passing within their communication range. With the help of the designed miniature antenna placed on the RSU, they can broadcast traffic advisories, road conditions, and emergency alerts to improve situational awareness for nearby vehicles in the DSRC range. Moreover, they can collect data from multiple vehicles within their coverage area with the help of this antenna and aggregate it. RSUs can store and process this information for further analysis. RSUs serve as access points to provide communication infrastructure support for VANETs. They establish communication links with vehicles passing nearby and relay messages between vehicles and other RSUs.

### Notations

The notation used in the proposed work are publicized in Table 1.

**Table 1:** List of notations.

Notations	Description
$\:G$	Multiplicative cyclic group
$\:P$	Generator of $\:G$
$\:q$	Large prime number
$\:\partial\:$	Secret private key of TA
$\:{puk}_{TA}$	Public key of TA
$\:H\left(.\right)$	Hash function
$\:{OID}_{{v}_{i}}$,$\:{OID}_{{R}_{j}}$	Original id of the vehicle user and RSU
$\:{DID}_{{v}_{i}},{DID}_{{R}_{j}}$	Dummy id of the vehicle user and RSU
$\:{\delta\:}_{i}$,$\:{\delta\:}_{j},{\lambda\:}_{j}$	Random number chosen by the vehicle user and RSU
$\:{r}_{i}$,$\:{r}_{j}$	Random numbers chosen by TA
$\:{sk}_{{v}_{i}},{sk}_{{R}_{j}}$	Secret key of vehicle user and RSU
$\:{puk}_{{v}_{i}},{puk}_{{R}_{j}}$	Public key of vehicle user and RSU
$\:{\varpi\:}_{{v}_{i}},{\varpi\:}_{{R}_{j}}$	Verification parameter of vehicle user and RSU
$\:{T}_{i},{T}_{j},{tt}_{i}$	Timestamps
$\:{\sigma\:}_{j}$	Signature
$\:\psi\:$	One time secret key of vehicle user
$\:{Pu}_{v}$	One time public key of vehicle user
φ	Golden ratio
$\:{L}_{T}$	Typical length
$\:{L}_{P}$	Patch length
$\:m$	Miniaturization
$\:E,\:H$	Electric field, Magnetic field
$\:dBi$	Decibel isotropic
$\:{S}_{11}$	Return loss

## Proposed scheme

Two key issues with VANET communications are addressed by the proposed certificateless aggregate signature scheme: (1) the requirement for effective authentication in high-mobility, high-traffic environments, and (2) the need to reduce computational and communication overhead to enable real-time operations. By removing the certificate administration load associated with conventional PKI-based systems, certificateless cryptography lowers communication latency and complexity. In vehicle environments with limited bandwidth, aggregate signatures greatly reduce communication cost by combining several authentication messages into a single, compact signature. Moreover, cryptographic operations are accelerated by hardware-level implementation through antenna integration, which lowers latency and permits quicker authentication appropriate for high-speed vehicle communication. The proposed scheme consists of the following stages: Initial registration, miniature antenna design for RSU, and OBU of the vehicle user for DSRC communication, authentication, signature generation, verification, and aggregate signature validation.

### TA initialization

To begin, the secret private key is chosen at random by the TA and is denoted as $\:\partial\:\:\in\:{Z}_{q}^{\text{*}}$. Moreover, $\:G$ is a multiplicative cyclic group of order $\:q$ selected by TA. Assume that $\:p$ is $\:G$’s generator. The TA public key is calculated as $\:{puk}_{TA}=\partial\:P$. Further, TA chooses one way hash functions as $\:H\left(.\right):\{\text{1,0}{\}}^{*}\to\:\{\text{1,0}{\}}^{{l}_{n}}$and sets the public parameters $\:pp=(q,p,G,{puk}_{TA},H)$ in the exposed medium.

### Vehicle user $\:\left({V}_{i}\right)$ registration

The $\:{V}_{i}$ chooses $\:{OID}_{{v}_{i}} \in \:{Z}_{q}^{\text{*}}$ as its original identity and picks a random number $\:{\delta\:}_{i} \in\:{Z}_{q}^{\text{*}}$ and calculates $\:{\eta\:}_{1}={\delta\:}_{i}P$. Later, it direct ($\:{OID}_{{v}_{i}},{\eta\:}_{1}$) to TA. TA computes $\:{\eta\:}_{2}=\partial\:{\eta\:}_{1}$ and the dummy identity for the patient as $\:{DID}_{{v}_{i}}={OID}_{{v}_{i}} \oplus H({\eta\:}_{1},{\eta\:}_{2},{puk}_{TA})$. Moreover, TA chooses $\:{r}_{i} \in \:{Z}_{q}^{\text{*}}$ and computes the following parameters

$$\:{A}_{i}={r}_{i}P$$

$$\:{B}_{i}=H({A}_{i},\:{puk}_{TA},{OID}_{{v}_{i}},{DID}_{{v}_{i}})$$

$$\:{C}_{i}=\left({r}_{i}+\partial\:\right)modq$$

$$\:{D}_{i}=({B}_{i}+{C}_{i})modq$$

$$\:{sk}_{{v}_{i}}=H\left({D}_{i}P,{A}_{i}\right)$$

$$\:{puk}_{{v}_{i}}={sk}_{{v}_{i}}{puk}_{TA}$$

$$\:{\varpi\:}_{{v}_{i}}=\partial\:{{DID}_{{v}_{i}}}^{-1}P$$

Here, $\:{\varpi\:}_{{v}_{i}}$ is the verification parameter for $\:{V}_{i}$. Later, TA provides $\:({sk}_{{v}_{i}},\:{puk}_{{v}_{i}},\:{\varpi\:}_{{v}_{i}},{DID}_{{v}_{i}})$ to the $\:{V}_{i}$ in a secure way.

### $\:{RSU}_{j}$ registration

$\:{RSU}_{j}$ chooses $\:{OID}_{{R}_{j}} \in\:{Z}_{q}^{\text{*}}$ as its original identity and picks a random number $\:{\delta\:}_{j} \in \:{Z}_{q}^{\text{*}}$ and calculates $\:{\mu\:}_{1}={\delta\:}_{j}P$. Later, it direct ($\:{OID}_{{R}_{j}},{\mu\:}_{1}$) to TA. TA calculates $\:{\mu\:}_{2}=\partial\:{\mu\:}_{1}$ and the dummy identity for the patient as $\:{DID}_{{R}_{j}}={OID}_{{R}_{j}} \oplus H({\mu\:}_{1},{\mu\:}_{2},{puk}_{TA})$. Moreover, TA chooses $\:{r}_{j} \in \:{Z}_{q}^{\text{*}}$ and computes the following parameters

$$\:{A}_{j}={r}_{j}P$$

$$\:{B}_{j}=H({A}_{j},\:{puk}_{TA},{OID}_{{R}_{j}},{DID}_{{R}_{j}})$$

$$\:{C}_{j}=\left({r}_{j}+\partial\:\right)modq$$

$$\:{D}_{j}=({B}_{j}+{C}_{j})modq$$

$$\:{E}_{j}={B}_{j}{puk}_{TA}$$

$$\:{sk}_{{R}_{j}}=H\left({D}_{j}P,{E}_{i}\right)$$

$$\:{puk}_{{R}_{j}}={sk}_{{R}_{j}}{puk}_{TA}$$

$$\:{K}_{j}={puk}_{{R}_{j}}+{E}_{j}$$

$\:{\varpi\:}_{{R}_{j}}=\partial\:{{DID}_{{R}_{j}}}^{-1}P$, Here $\:{\varpi\:}_{{R}_{j}}$ is the verification parameter of $\:{RSU}_{j}$. Later, TA provides $\:({sk}_{{R}_{j}},\:{puk}_{{R}_{j}},\:{\varpi\:}_{{R}_{j}},{DID}_{{R}_{j}},{K}_{j})$ to $\:{RSU}_{j}$ in a secure way.

### Miniature antenna design for DSRC communication

Our suggested design views the antenna module not as an independent communication component, but as a facilitator of safe and effective communication. In particular, the VANET architecture’s cryptographic layer proposes a signature aggregation approach, which is integrated with the small antenna. Unlike other parts of the VANET security design, the antenna module actively contributes. Incorporating a complementary relationship between hardware architecture and cryptographic protocol execution, its design has a direct impact on the spatial aspect of secure communication. The VANET system is made more trustworthy and resilient with this multi-layered approach. Communication in Vehicular Ad-Hoc Networks (VANETs) must be secure and dependable for all applications, but notably those that communicate safety-critical information. The cryptographic algorithms secure the data level, but the physical layer is crucial for geographic secrecy, eavesdropping resistance, and jamming robustness. To enhance the secure communication in DSRC, a novel miniature antenna is designed. This antenna is placed in the OBU of the $\:{V}_{i}$and $\:{RSU}_{j}$. An efficient transfer of LBI takes place between $\:{V}_{i}$and $\:{RSU}_{j}$ in DSRC at a frequency of 5.9 GHz with the help of this newly designed antenna. This antenna is constructed using concentric octagonal rings that are developed from the ‘Golden ratio’ principle. A couple of slots have been strategically positioned relative to the centre of the concentric ring-shaped design to achieve resonance at the necessary frequency of 5.90 GHz. At a frequency of 5.90 GHz in the DSRC band, the antenna can provide full coverage of the impedance bandwidth, a good level of gain, little cross-polarization discrimination, and great radiation efficiency.The formation details of the miniature antenna and its complete embodiment are explained as trails.

#### Formation

**Fig. 2:**
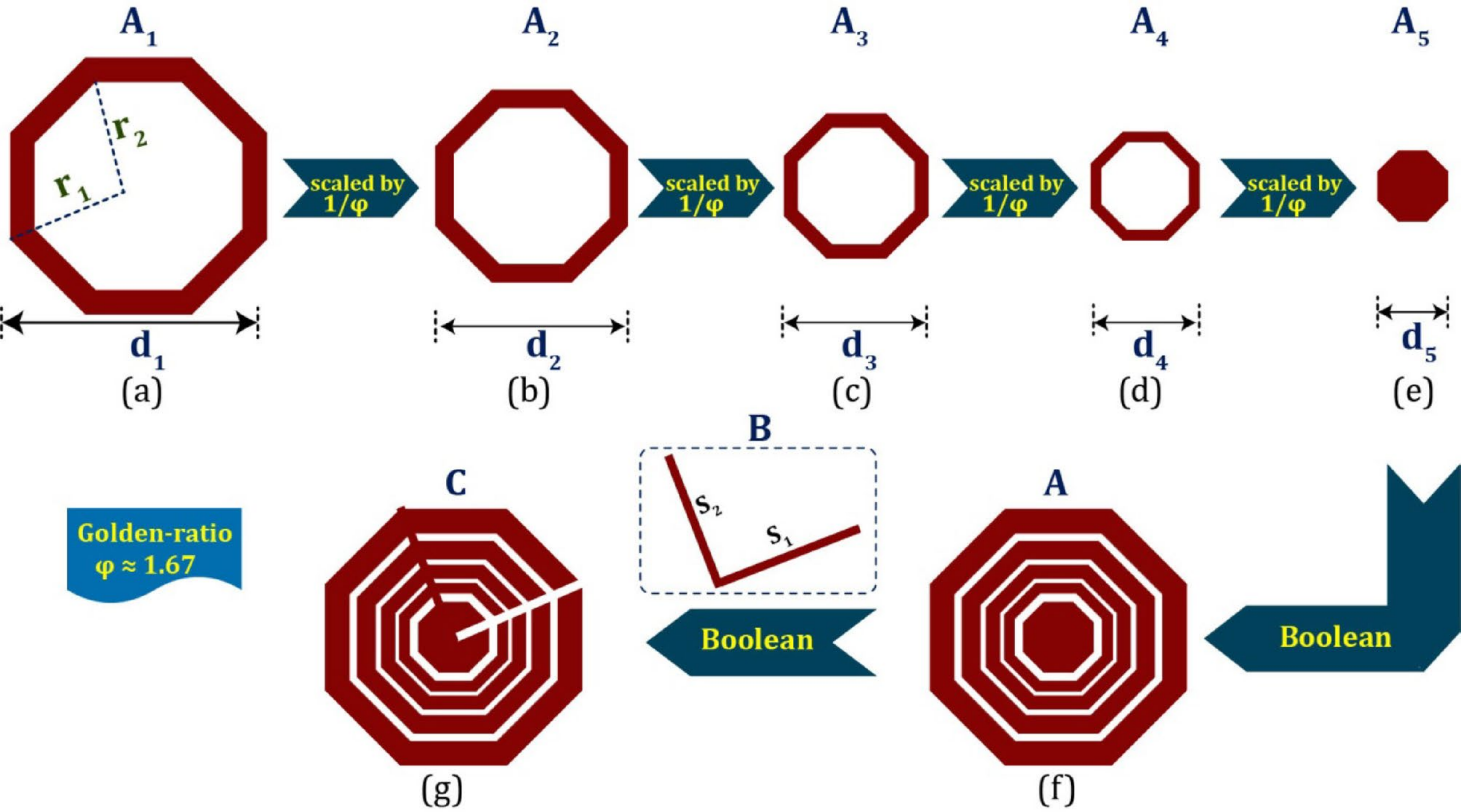
Stepwise formation of the proposed geometry (r_1_ = 7.0, r_2_ = 6.5, d_1_ = 14.0; unit: mm).

The proposed octagonal concentric-ring-shaped slotted structure has been derived from four octagonal rings, a solid octagon, and a couple of thin rectangular slots, respectively. The detailed formation has been carried out graphically in Fig. 1. Initially, an octagonal ring **A**_**1**_has been considered where it’s outer and inner ring radiuses are indicated as r_1_ and r_2_, respectively. The overall length of the initial ring is fixed as d1, as conferred in Fig. 2a. In the next step, the structure **A**_**1**_ is scaled down by 0.60 (1/Golden-ratio) and obtained the scaled octagonal ring A2 as depicted in Fig. 2b. In a similar way, the structure **A**_**2**_ is two times scaled down by 0.60 and obtained the structures **A**_**3**_ and **A**_**4**_, as depicted in Fig. 2c,d, respectively. Later, another solid octagonal structure **A**_**5**_, is introduced, whose radius is fixed as a 0.60 scaled version of the radius of **A**_**4**_, and it is shown in Fig. 2e. All those five octagonal geometries are Boolean and obtained the concentric octagonal-ring-shaped geometry **A** as depicted in Fig. 2f. This kind of Boolean structure enhances the overall electrical length, which helps to achieve decent gain at the desired frequency for particular applications. Further, an inclined V-shaped structure **B**, which consists of a couple of slot, has been introduced. The structure **B** is placed in the optimal position of the structure A. To make the connectivity among every ring, the thin-slot S_2_ has been added. In order to resonate the structure at the desired frequency, slot S_1_ has been etched from structure A and obtained the proposed intended geometry **C** as depicted in Fig. 2g. The associated dimension of all the sub-geometries has been mentioned in the caption of Fig. 1. To achieve effective frequency transmission in the DSRC band (5.85–5.925 GHz), especially for Vehicular Ad-hoc Networks (VANETs), this study primarily focused on proposing a new and compact antenna configuration based on the ‘Golden-ratio’ concept. Present findings are based only on current state-of-the-art full-wave electromagnetic simulations performed in CST Microwave Studio. We understand that manufacturing tolerances, ambient factors, and integration issues can cause real-world implementation to deviate from predicted behavior. Therefore, it is now explicitly stated in the updated text that our current and future work includes fabricating hardware prototypes and conducting empirical validation. Before moving on to prototype manufacturing and testing, offering the idea with thorough theoretical analysis and simulated validation is essential. This is particularly true when introducing a new design technique.

The overall length of each octagonal structure is inversely proportional to the ‘Golden-ratio (φ)’ and it can be determined as follows:

1 $$\:{d}_{i+1}=\frac{1}{\phi\:}{d}_{i}\:\text{w}\text{h}\text{e}\text{r}\text{e},\:i=\text{1,2},\text{3,4},5.$$

**Fig. 3:**
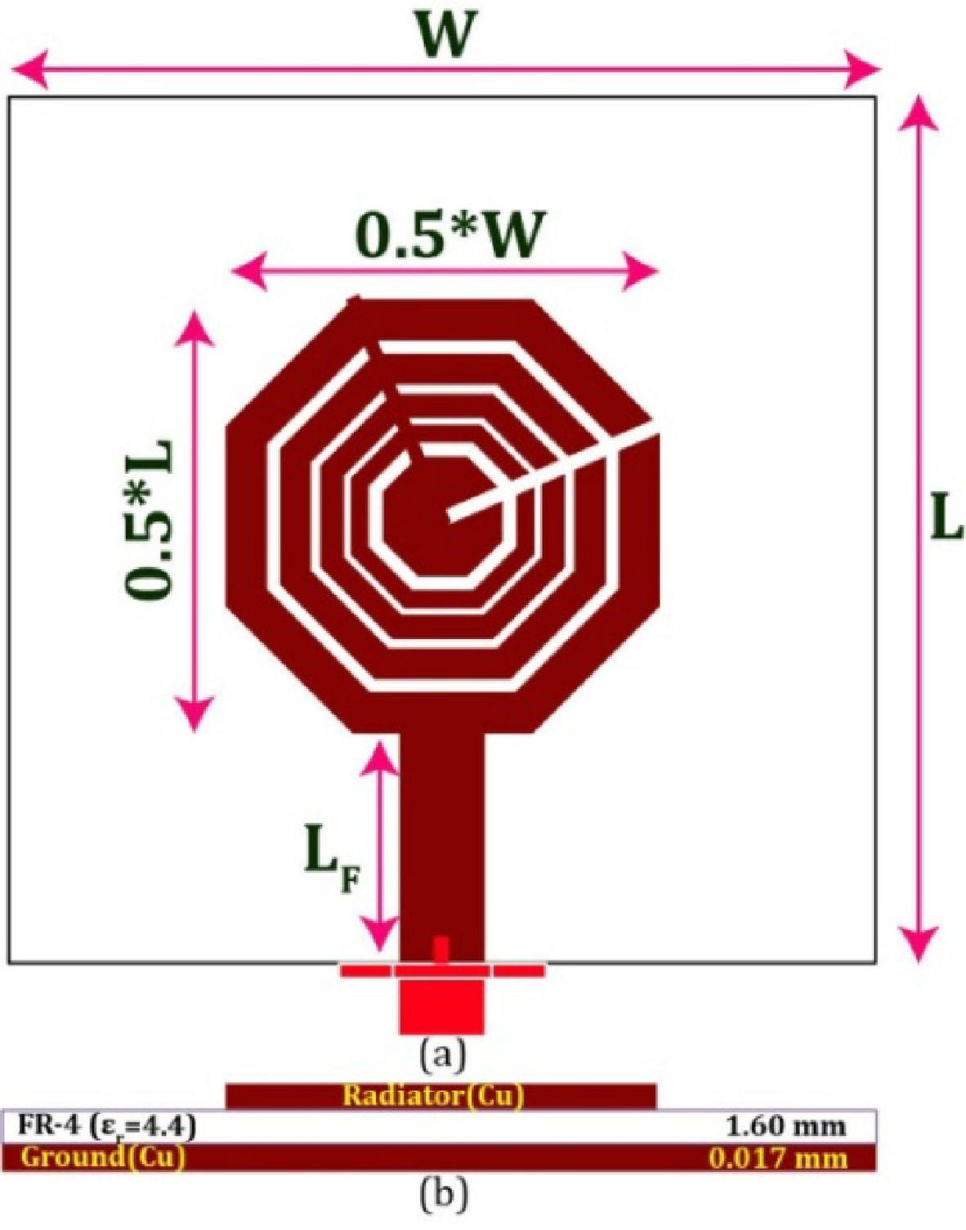
Proposed printed radiator (W = L = 28.0, L_F_ = 7.0; unit: mm).

#### Complete embodiment

Structure C, derived from the sequential development of the suggested structure, has been imprinted onto a printed circuit board (PCB) laminated substrate composed of FR-4 material (with a relative permittivity of 4.3). The substrate material has a constant height of 1.60 mm, whereas the ground and radiator layers have a fixed thickness of 0.017 mm. The structure is excited using a 50Ω source by including a strip-line feed. Figure 3a shows the antenna’s geometry’s frontal perspective. The opposite surface of the substrate is used as a comprehensive ground layer. The layer-wise structure is seen in Fig. 3(b). The antenna was developed and simulated using CST Microwave Studio (v2019).

The following are some of the most salient aspects of our proposed antenna module that we concentrated on:

• Reducing spurious modes to improve bandwidth uniformity,
• Keeping the device small, this is important for integration with vehicles.
• Reducing cross-coupling in array designs;
• Enhancing impedance matching over a broader frequency range.

#### Compactness

The conventional printed antenna, fabricated using FR-4, often has a length denoted as $\:{L}_{T}$, measuring 15.62 mm, enabling its operation at a frequency of 5.90 GHz. The dimension denoted as $\:{L}_{P}$, representing the length of the recently developed structure, is measured to be 14.00 mm, as seen in Fig. 3a. The degree of miniaturization ($\:m$) may be determined using the following formula:

$$\:m=\left(\frac{{{L}_{T}}^{2}-{{L}_{P}}^{2}}{{{L}_{T}}^{2}}\right)\times\:100\%$$

Hence, the intended antenna’s size has been miniaturized by 19.67%, touted as an innovation.

### $\:{V}_{i}$ authentication by $\:{RSU}_{j}$

Before transferring the information between the $\:{RSU}_{j}$ and $\:{V}_{i}$, both of them should be mutually authenticated. When the $\:{V}_{i}$ enters the $\:{RSU}_{j}$ region, the vehicle should be initially authenticated by $\:{RSU}_{j}$. To perform this authentication, $\:{V}_{i}$ computes the subsequent parameters

$$\:{\alpha\:}_{i}=H({puk}_{{v}_{i}},{puk}_{TA},{DID}_{{v}_{i}},{T}_{i}$$)

$$\:{\beta\:}_{i}=\left({sk}_{{v}_{i}}+{DID}_{{v}_{i}}\right){{\alpha\:}_{i}}^{-1}mod\:q$$

$$\:{\xi\:}_{{v}_{i}}={sk}_{{v}_{i}}{\varpi\:}_{{v}_{i}}$$

After scheming these parameters, $\:({\alpha\:}_{i},{\beta\:}_{i},{T}_{i},{DID}_{{v}_{i}},{\varpi\:}_{{v}_{i}},{\xi\:}_{{v}_{i}})$ are provided to the $\:{RSU}_{j}$. Based on these parameters,$\:{RSU}_{j}$ initially verifies timestamp, then calculates $\:{{\alpha\:}_{i}}^{*}=H({puk}_{{v}_{i}}$,$\:{puk}_{TA},{DID}_{{v}_{i}},{T}_{i}$). If $\:{{\alpha\:}_{i}}^{*}={\alpha\:}_{i}$, then the $\:{RSU}_{j}$ calculates $\:{\gamma\:}_{j}={{\alpha\:}_{i}}^{*}{\beta\:}_{i}mod\:q$. Finally, the $\:{RSU}_{j}$ validates the condition $\:{\gamma\:}_{j}{\varpi\:}_{{v}_{i}}={\xi\:}_{{v}_{i}}+\:{puk}_{TA}$, if the condition indulges, then the $\:{V}_{i}$ is authenticated.

*Proof of validation*

$$\:{\gamma\:}_{j}{\varpi\:}_{{v}_{i}}=\left({{\alpha\:}_{i}}^{*}{\beta\:}_{i}\right){\varpi\:}_{{v}_{i}}$$

$$\:={{\alpha\:}_{i}}^{*}\left({sk}_{{v}_{i}}+{DID}_{{v}_{i}}\right){{\alpha\:}_{i}}^{-1}{\varpi\:}_{{v}_{i}}$$

$$\:=\left({sk}_{{v}_{i}}+{DID}_{{v}_{i}}\right){\varpi\:}_{{v}_{i}}$$

$$\:={sk}_{{v}_{i}}{\varpi\:}_{{v}_{i}}+{DID}_{{v}_{i}}{\varpi\:}_{{v}_{i}}$$

$$\:={\xi\:}_{{v}_{i}}+{DID}_{{v}_{i}}\partial\:{{DID}_{{v}_{i}}}^{-1}P$$

$$\:={\xi\:}_{{v}_{i}}+\partial\:P$$

$$\:={\xi\:}_{{v}_{i}}+{puk}_{TA}$$

### $\:{RSU}_{j}$authentication by $\:{V}_{i}$

The same above procedure is repeated and finally the $\:{V}_{i}$ receives $\:({\alpha\:}_{j},{\beta\:}_{j},{DID}_{{R}_{i}},{T}_{j},\:{\varpi\:}_{{R}_{i}},{\xi\:}_{{R}_{i}})$ from $\:{RSU}_{j}$ and the $\:{V}_{i}$initially checks the freshness of timestamp, later checks $\:{{\alpha\:}_{j}}^{*}={\alpha\:}_{j}$. If gratified, then the $\:{V}_{i}$ computes $\:{\gamma\:}_{i}={{\alpha\:}_{j}}^{*}{\beta\:}_{j}modq$. Finally, the $\:{V}_{i}$ validates the condition $\:{\gamma\:}_{i}{\varpi\:}_{{R}_{j}}={\xi\:}_{{R}_{j}}+\:{puk}_{TA}$, if the condition indulges, then the $\:{RSU}_{j}$ is authenticated. Once mutual authentication is successfully executed, message authentication follows.

### Message authentication and signature generation

To share the LBI with the use of the designed antenna, $\:{RSU}_{j}$ picks a random number $\:{\lambda\:}_{j} \in \:{Z}_{q}^{\text{*}}$ and computes the following terms

$$\:{X}_{j}={\lambda\:}_{j}{puk}_{TA}$$

$$\:{Y}_{j}=H({X}_{j},LBI,{DID}_{{R}_{j}},\:{tt}_{j})$$

$$\:{Z}_{j}={Y}_{j}\left({sk}_{{R}_{i}}+H\left({K}_{j}\right)\right)+{\lambda\:}_{j}$$

$$\:{L}_{j}={puk}_{{R}_{i}}+H\left({K}_{j}\right){puk}_{TA}$$

Based on the above terms, the signature is computed as $\:{\sigma\:}_{j}=({X}_{j},{Z}_{j})$. Finally,$\:{RSU}_{j}$ shares

$\:(LBI,\:{tt}_{j},{\sigma\:}_{j},{L}_{j})$with the help of the designed antenna in the DSRC range at 5.9 GHz frequency to the $\:{V}_{i}$.

### Signature verification

$\:{V}_{i}$ checks the freshness of $\:{tt}_{j}$, later checks $\:{Z}_{j}{puk}_{TA}={Y}_{j}{L}_{j}+{X}_{j}$, where $\:{Y}_{j}^{*}=H({X}_{j},LBI,{DID}_{{R}_{j}},\:{tt}_{j})$. If the condition is verified, single signature is verified and the LBI is accepted by the$\:{V}_{i}$.

*Proof of validation*

$$\:{Z}_{j}{puk}_{TA}=[{Y}_{j}\left({sk}_{{R}_{i}}+H\left({K}_{j}\right)\right)+{\lambda\:}_{j}]{puk}_{TA}$$

$$\:={Y}_{j}\left({sk}_{{R}_{i}}{puk}_{TA}+H\left({K}_{j}\right){puk}_{TA}\right)+{\lambda\:}_{j}{puk}_{TA}$$

$$\:={Y}_{j}({puk}_{{R}_{i}}+H\left({K}_{j}\right){puk}_{TA})\:+{X}_{j}$$

$$\:={Y}_{j}{L}_{j}+{X}_{j}$$

#### Aggregate signature validation

If $\:n$ number of $\:{LBI}_{j}$ say $\:\left({LBI}_{1},{LBI}_{2}.{LBI}_{n}\right)$ are send by the $\:{RSU}_{j}$ to the $\:{V}_{i}$ at the same instant. Then, all the $\:{LBI}_{j}$ should be verified by the $\:{V}_{i}$ at the same time. This is possible with the help of the designed antenna which have a larger bandwidth. To perform this, $\:{V}_{i}$ chooses the one-time secret key $\:\psi\:\: \in \:{Z}_{q}^{\text{*}}$ and calculates the one-time public key $\:{Pu}_{v}=\psi\:{puk}_{TA}$. Later,$\:{V}_{i}$ sends $\:{Pu}_{v}$ to $\:{RSU}_{j}$ using the designed miniature antenna present on the OBU of the $\:{V}_{i}$. Based on the received parameter, $\:{RSU}_{j}$ computes the following parameters$\:{\Omega\:}=H\left({Z}_{1}{Pu}_{v}\left|\left|{Z}_{2}{Pu}_{v}\right|\right|{Z}_{3}{Pu}_{v}\right||\dots\:{Z}_{j}{Pu}_{v})$, $\:X=\sum\:_{j=1}^{n}{X}_{j},\:Y=\sum\:_{j=1}^{n}{Y}_{j}$,$\:Z=\sum\:_{j=1}^{n}{Z}_{j}$, $\:L=\sum\:_{j=1}^{n}{L}_{j}$and computes the aggregate signature as $\:\sigma\:=\left(X,Z\right)$. Finally, $\:\sigma\:$ and $\:{(LBI}_{j}$,$\:{tt}_{j},\:{\Omega\:},L)$ are send the $\:{V}_{i}.$ with the help of the designed antenna.

The $\:{V}_{i}$ checks the condition$\:\sum\:_{j=1}^{n}{Z}_{j}{puk}_{TA}=\sum\:_{j=1}^{n}({Y}_{j}{L}_{j}+{X}_{j})=\sum\:_{j=1}^{n}{{\Lambda\:}}_{j}$ Where $\:{{\Lambda\:}}_{j}=({Y}_{j}{L}_{j}+{X}_{j})$ and $\:{\Omega\:}$. If both the conditions are validated, then the $\:{LBI}_{j}$ are accepted by the $\:{V}_{i}$.

*Proof of validation*

$$\:\sum\:_{j=1}^{n}{Z}_{j}{puk}_{TA}=\sum\:_{j=1}^{n}\left[{Y}_{j}\left({sk}_{{R}_{i}}+H\left({K}_{j}\right)\right)+{\lambda\:}_{j}\right]{puk}_{TA}$$

$$\:=\sum\:_{j=1}^{n}{Y}_{j}\left({sk}_{{R}_{i}}{puk}_{TA}+H\left({K}_{j}\right){puk}_{TA}\right)+\sum\:_{j=1}^{n}{\lambda\:}_{j}{puk}_{TA}$$

$$\:=\sum\:_{j=1}^{n}{Y}_{j}({puk}_{{R}_{i}}+H\left({K}_{j}\right){puk}_{TA})\:+\sum\:_{j=1}^{n}{X}_{j}$$

$$\:=\sum\:_{j=1}^{n}{Y}_{j}{L}_{j}+\sum\:_{j=1}^{n}{X}_{j}=\sum\:_{j=1}^{n}{{\Lambda\:}}_{j}$$

$$\:=YL+X$$

$$\:{\Omega\:}=H\left(\psi\:{{\Lambda\:}}_{1}\left|\left|\psi\:{{\Lambda\:}}_{2}\right|\right|\psi\:{{\Lambda\:}}_{2}\right||\dots\:\psi\:{{\Lambda\:}}_{3})$$

$$\:=H\left({Z}_{1}\psi\:{puk}_{TA}\left|\left|{Z}_{2}\psi\:{puk}_{TA}\right|\right|{Z}_{3}\psi\:{puk}_{TA}\right||\dots\:{Z}_{j}\psi\:{puk}_{TA})$$

$$\:=H\left({Z}_{1}{Pu}_{v}\left|\left|{Z}_{2}{Pu}_{v}\right|\right|{Z}_{3}{Pu}_{v}\right||\dots\:{Z}_{j}{Pu}_{v})$$

## Security analysis

Security analysis of the proposed scheme is evaluated in terms of informal and formal analysis.

### Resist to impersonation attack

When the legitimate vehicle user or RSU is impersonated, there may be a possibility of this kind of attack. Impersonation can be performed by gaining the access of the vehicle user or RSUs credentials. This attack can be also performed by intercepting and manipulating the data communication between the entities. To impersonate as a $\:{V}_{i}$, the attacker should compute the following parameters $\:({\alpha\:}_{i},{\beta\:}_{i},{DID}_{{v}_{i}},{\varpi\:}_{{v}_{i}},{\xi\:}_{{v}_{i}})$ and provide it to the $\:{RSU}_{j}$. Here, $\:{\alpha\:}_{i}$ and $\:{\beta\:}_{i}$ are computed based on $\:{DID}_{{v}_{i}}$ and $\:{sk}_{{v}_{i}}$, which are provided by TA during initial offline registration. Further, $\:{\varpi\:}_{{v}_{i}}$ is the verification parameter calculated based on the secret key of TA. Thus, it is not possible for an intruder to compute all these parameters for validation. Similarly, to impersonate as valid $\:{RSU}_{j}$, the corresponding $\:{RSU}_{j}$ should compute the subsequent parameters $\:({\alpha\:}_{j},{\beta\:}_{j},{DID}_{{R}_{i}},{\varpi\:}_{{R}_{i}},{\xi\:}_{{R}_{i}})$ and provide it to $\:{V}_{i}$ for validation. But computing the above parameters requires the same difficulty similar to$\:{V}_{i}$ validation. Thus, impersonating as $\:{V}_{i}$ or $\:{RSU}_{j}$ is impossible in the suggested protocol.

### Resist to fake data attack

This attack is possible, when an intruder completely sends a fake LBI in place of genuine information. But the suggested work is secure against the fake data assault. Only after successful authentication of $\:{V}_{i}$, $\:{RSU}_{j}$ sends the LBI to the $\:{V}_{i}.\:$Moreover, during the transfer of LBI, the $\:{RSU}_{j}$ sends the LBI with signature ($\:{\sigma\:}_{j}$) attachment. So, to replace the LBI with fake data, the $\:{\sigma\:}_{j}$ attached to LBI also should be modified. But modifying the $\:{\sigma\:}_{j}$ is impossible, as it involves $\:{X}_{j},{Z}_{j}$ parameters. Here, $\:{Z}_{j}$ is computed as $\:{Z}_{j}={Y}_{j}\left({sk}_{{R}_{i}}+H\left({K}_{j}\right)\right)+{\lambda\:}_{j}$, which involves the secret key of $\:{RSU}_{j}$, and the $\:{DID}_{{R}_{j}}$for the computation of $\:{K}_{j}$. Thus, it is hard to compute the $\:{Z}_{j}$ and to modify or recreate the$\:{\sigma\:}_{j}$ to perform fake data attack.

### Resist to data modification attack

A portion or the complete LBI is modified, it leads to data modification attack. The validation of the LBI fails, if LBI is modified or altered and send to the $\:{V}_{i}$ by $\:{RSU}_{j}$. Since the $\:{V}_{i}$ checks the condition $\:{Z}_{j}{puk}_{TA}={Y}_{j}{L}_{j}+{X}_{j}$. In order to validate the condition, the parameter $\:{Z}_{j}$ in the signature should be computed. But the computation of $\:{Z}_{j}$ involves $\:{sk}_{{R}_{i}}$ and $\:{K}_{j}$, which are hard to be computed. Since $\:{sk}_{{R}_{i}}$ and $\:{K}_{j}$ which involves $\:{DID}_{{R}_{j}}$ are provided to $\:{RSU}_{j}$ by TA during initial offline registration in a secure way. Its not possible for an intruder to track these parameters and to compute it. Thus, the above condition fails, and the proposed scheme is secure against data modification attack.

### Resist to repudiation attack

It is a type of cyberattack that aims to deny the authenticity of a message sent by another vehicle or RSU (i.e.,) the $\:{RSU}_{j}$ denies after sending the LBI to the $\:{V}_{i}$. But the suggested protocol is free from repudiation assault, since the $\:{RSU}_{j}$ and $\:{V}_{i}$ are registered with the TA. Only the authenticated and legitimate entities are allowed to participate in the network. As a result, the registered and authenticated $\:{RSU}_{j}$ and $\:{V}_{i}$ cannot deny after sending the data. In this work, the authenticity of the $\:{V}_{i}$ is validated by checking the condition $\:{\gamma\:}_{j}{\varpi\:}_{{v}_{i}}={\xi\:}_{{v}_{i}}+\:{puk}_{TA}$. Similarly, the authenticity of the $\:{RSU}_{j}$ is verified by validating the condition $\:{\gamma\:}_{i}{\varpi\:}_{{R}_{j}}={\xi\:}_{{R}_{j}}+\:{puk}_{TA}$. Only after mutual authentication between the entities, both the $\:{RSU}_{j}$ and $\:{V}_{i}$ are allowed to exchange the data between them. The authenticated entity cannot repudiate after transferring the data, thus the work is free from repudiation assault.

### Anonymity and privacy preserving

The entities use their pseudonyms to share data with one another. Thus, the $\:{RSU}_{j}$ and $\:{V}_{i}$ identities are kept private. The intruders gain no knowledge from the fake identity’s capture. As a result, anonymity is preserved. Furthermore, a signature is appended to the LBI during transmission. This protects the critical, private information that is only shared with the $\:{V}_{i}$. As an outcome, the proposed work maintains privacy.

### Resist to replay attack

The attacker intercepts the communication, modifies LBI, and sends it again. This results in replay assault. This can be overcome by adding a timestamp to the transferred LBI. In this work, timestamp is used during mutual authentication and signature generation. A time stamp is appended during mutual authentication between the $\:{RSU}_{j}$ and $\:{V}_{i}$ to avoid reply assault. Furthermore, signature along with LBI, timestamp issent to the $\:{V}_{i}$ for validation. Initially, timestamp is verified, later signature is validated. Thus, the entity first looked for the timestamp after getting the data. The entity will check the necessary condition to authenticate the message’s authenticity (LBI) if the timestamp is verified, meaning that the content was received within the allotted time limit. Thus, the recommended work is hence free from reply assault.

### Resist to sybil attack

In sybil attack, an adversary creates multiple fake identities and disrupt the functionality of the system. But in our scheme, a unique pseudo-identity is attached to the signature and verified through signature verification scheme. The vehicle user verifies $\:{Z}_{j}{puk}_{TA}={Y}_{j}{L}_{j}+{X}_{j}$, where $\:{Y}_{j}=H({X}_{j},LBI,{DID}_{{R}_{j}},\:{tt}_{j})$. If the condition is verified, then the single user identity is validated. Since the creation of dummy identity involves the private key of TA, it prevents the adversary from generating multiple valid pseudo-identities.

### Resist to man-in-the-middle attack (MITM)

In this work, mutual authentication between vehicles and RSU occurs by validating the condition $\:{\gamma\:}_{j}{\varpi\:}_{{v}_{i}}={\xi\:}_{{v}_{i}}+\:{puk}_{TA}$ and $\:{\gamma\:}_{i}{\varpi\:}_{{R}_{j}}={\xi\:}_{{R}_{j}}+\:{puk}_{TA}$. If an intruder, tries to pretend to be a valid entity, he should satisfy the above condition. However, the equations involve the public key of TA, it is hard for an intruder to satisfy it. Further, the condition will not be satisfied if the parameters are changed during the mutual authentication process. As a result, the proposed work is secure against MITM attack.

### Resist to deniel of service (DOS) attack

In this work, group of messages are validated using aggregate signature validation scheme. Aggregate signature significantly reduces the number of individual verification operation required. As a result, even under high volume of messages, the RSUs can verify the batch message effectively. To conclude, the proposed protocol is resistant against DOS attack.

#### Formal security analysis

Formal security is achieved using Burrows, Abadi, and Needham (BAN) logic^29^. For mutual authentication and signature validation, the following goals and assumptions are made and the statements are enumerated as follows.

$$\:Goal\_1:{V}_{i}|\equiv\:{V}_{i}\underleftrightarrow{LBI}{R}_{j}$$

$$\:Goal\_2:{V}_{i}|\equiv\:{R}_{j}|\equiv\:{V}_{i}\underleftrightarrow{LBI}{R}_{j}$$

$$\:Goal\_3:{R}_{j}|\equiv\:{V}_{i}\underleftrightarrow{LBI}{R}_{j}$$

$$\:Goal\_4:{R}_{j}|\equiv\:{V}_{i}|\equiv\:{V}_{i}\underleftrightarrow{LBI}{R}_{j}$$

The following idealization is performed for mutual authentication and LBI transfer between$\:{V}_{i}\underleftrightarrow{LBI}{R}_{j}.$

$$\:{M}_{1}:{V}_{i}\to\:{R}_{j}:\{{\alpha\:}_{i},{\beta\:}_{i},{DID}_{{v}_{i}},{\varpi\:}_{{v}_{i}},{\xi\:}_{{v}_{i}}\}$$

$$\:{M}_{2}:{R}_{j}\to\:{V}_{i}:\{{\alpha\:}_{j},{\beta\:}_{j},{DID}_{{R}_{i}},\:{\varpi\:}_{{R}_{i}},{\xi\:}_{{R}_{i}}\}$$

$$\:{M}_{2}:{R}_{j}\to\:{V}_{i}:\{LBI,\:{\sigma\:}_{j},{L}_{j}\}$$

The security assumptions are made based on the mutual authentication and signature validation:

$$\:{A}_{1}:{R}_{j}|\equiv\:\#\{{\alpha\:}_{i},{\beta\:}_{i},{DID}_{{v}_{i}},{\varpi\:}_{{v}_{i}},{\xi\:}_{{v}_{i}}\}$$

$$\:{A}_{2}:\:{R}_{j}|\equiv\:{V}_{i}=>{M}_{1}$$

$$\:{A}_{3}:{V}_{i}|\equiv\:\#\:\{{\alpha\:}_{j},{\beta\:}_{j},{DID}_{{R}_{i}},\:{\varpi\:}_{{R}_{i}},{\xi\:}_{{R}_{i}}\}$$

$$\:{A}_{4}:\:{V}_{i}|\equiv\:{R}_{j}=>{M}_{2}$$

$$\:{A}_{5}:{V}_{i}|\equiv\:\#\:\{{\lambda\:}_{j}\}$$

$$\:{A}_{6}:\:{V}_{i}|\equiv\:{R}_{j}=><{\sigma\:}_{j}>{X}_{j},{Z}_{j}$$

$$\:{A}_{7}:\:{V}_{i}|\equiv\:<LBI>{\sigma\:}_{j}$$

$$\:{A}_{8}:\:{V}_{i}|\equiv\:{R}_{j}=><LBI>{\sigma\:}_{j}$$

$$\:{A}_{9}:\:{V}_{i}|\equiv\:{R}_{j}=>{M}_{3}$$

The mutual authentication between $\:{V}_{i}$ and $\:{R}_{j}$ based on the rules^29^ and above mentioned assumptions are stated as follows.

From $$\:{M}_{1}$$

$$\:S1:{R}_{j} \triangleleft \{{\alpha\:}_{i},{\beta\:}_{i},{DID}_{{v}_{i}},{\varpi\:}_{{v}_{i}},{\xi\:}_{{v}_{i}}\}$$

$$\:S2:{R}_{j}|\equiv\:{V}_{i}|\sim\:\{{\alpha\:}_{i},{\beta\:}_{i},{DID}_{{v}_{i}},{\varpi\:}_{{v}_{i}},{\xi\:}_{{v}_{i}}\}$$

Based on $\:{A}_{1}$ and $\:S2$

$$\:S3:{R}_{j}|\equiv\:{V}_{i}|\equiv\:\{{\alpha\:}_{i},{\beta\:}_{i},{DID}_{{v}_{i}},{\varpi\:}_{{v}_{i}},{\xi\:}_{{v}_{i}}\}$$

$$\:S4:{R}_{j}|\equiv\:\left\{{\alpha\:}_{i},{\beta\:}_{i},{DID}_{{v}_{i}},{\varpi\:}_{{v}_{i}},{\xi\:}_{{v}_{i}}\right\}={\gamma\:}_{j}{\varpi\:}_{{v}_{i}}={\xi\:}_{{v}_{i}}+\:{puk}_{TA}\text{b}\text{a}\text{s}\text{e}\text{d}\:\text{o}\text{n}\:{A}_{2}$$

$$\:S5:{V}_{i}\triangleleft \{{\alpha\:}_{j},{\beta\:}_{j},{DID}_{{R}_{i}},\:{\varpi\:}_{{R}_{i}},{\xi\:}_{{R}_{i}}\}$$

$$\:S6:{V}_{i}|\equiv\:{R}_{j}|\sim\:({\alpha\:}_{j},{\beta\:}_{j},{DID}_{{R}_{i}},\:{\varpi\:}_{{R}_{i}},{\xi\:}_{{R}_{i}})$$

Based on $\:{A}_{3}$ and $\:S6$

$$\:S7:{V}_{i}|\equiv\:{R}_{j}|\equiv\:({\alpha\:}_{j},{\beta\:}_{j},{DID}_{{R}_{i}},\:{\varpi\:}_{{R}_{i}},{\xi\:}_{{R}_{i}})$$

$$\:S8:\:{V}_{i}|\equiv\:\left({\alpha\:}_{j},{\beta\:}_{j},{DID}_{{R}_{i}},\:{\varpi\:}_{{R}_{i}},{\xi\:}_{{R}_{i}}\right)={\gamma\:}_{i}{\varpi\:}_{{R}_{j}}={\xi\:}_{{R}_{j}}+\:{puk}_{TA}\:\text{b}\text{a}\text{s}\text{e}\text{d}\:\text{o}\text{n}\:{A}_{4}$$

Based on $\:{A}_{5}$ and $\:{A}_{6}$

$$\:S9: {V}_{i}\triangleleft {\left\{LBI\right\}}_{{\sigma\:}_{j}}$$

$$\:S10: {V}_{i}|\equiv\:{R}_{j}|\sim\:{\left\{LBI\right\}}_{{\sigma\:}_{j}}$$

$$\:S11:{V}_{i}\left|\equiv\:{R}_{j}\right|\equiv\:\#{\left\{LBI\right\}}_{{\sigma\:}_{j}}=<{\sigma\:}_{j}>{X}_{j},{Z}_{j}$$

Based on $\:{A}_{7}$ and$\:{A}_{8}$

$$\:S12:\:{V}_{i}|\equiv\:{\left\{LBI\right\}}_{{\sigma\:}_{j}}={Z}_{j}{puk}_{TA}={Y}_{j}{L}_{j}+{X}_{j}$$

Based on $\:S12$, the above-mentioned goals are accomplished.

## Results and discussion

Efficiency of the proposed protocol is analysed in terms of computational and communication overhead. Moreover, the designed antenna for efficient secure communication in VANET system is analysed based on the different parametric outcomes and it is described in the following sub-sections.

### Computational overhead

**Fig. 4:**
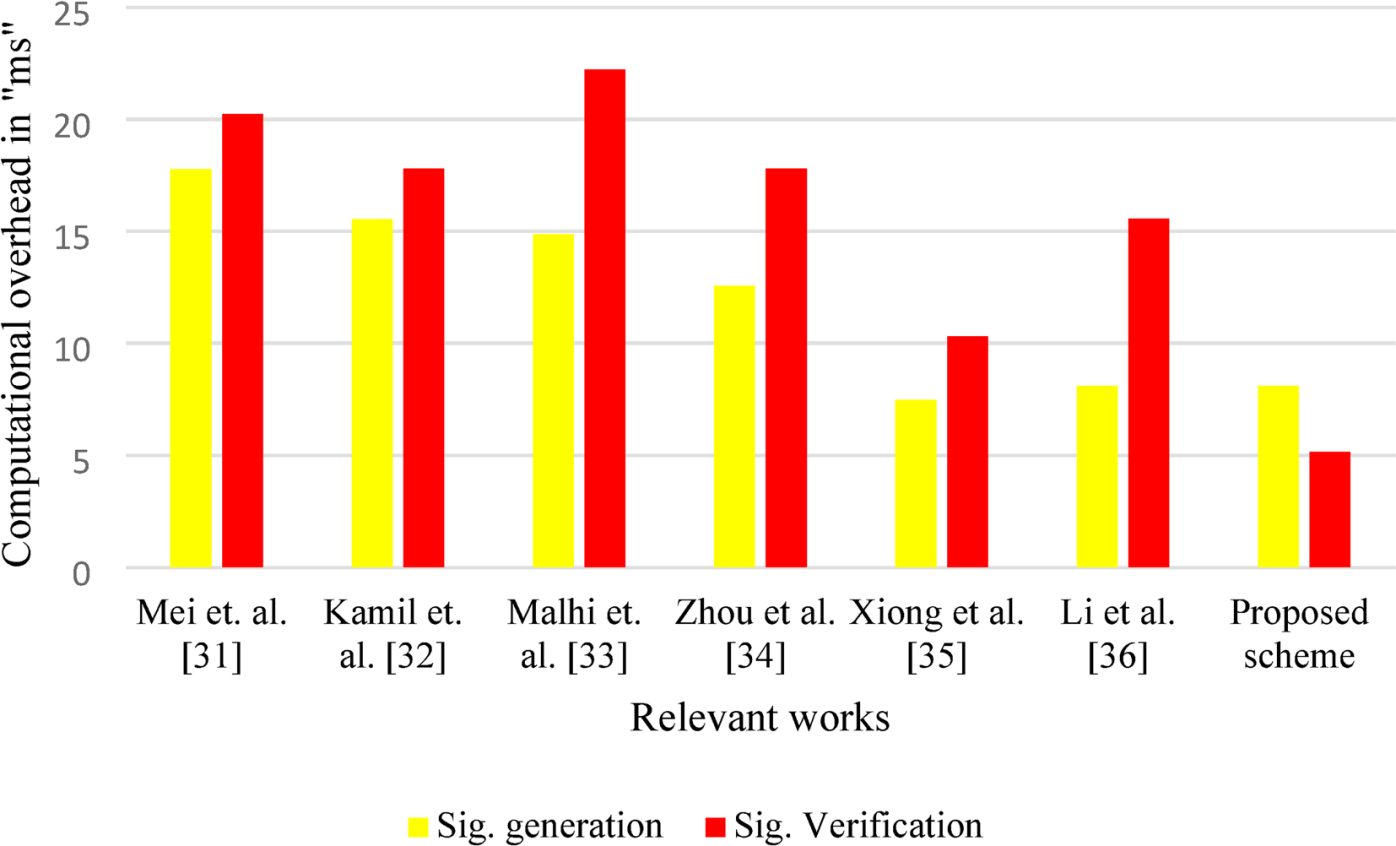
Computational overhead for signature generation and verification.

**Fig. 5:**
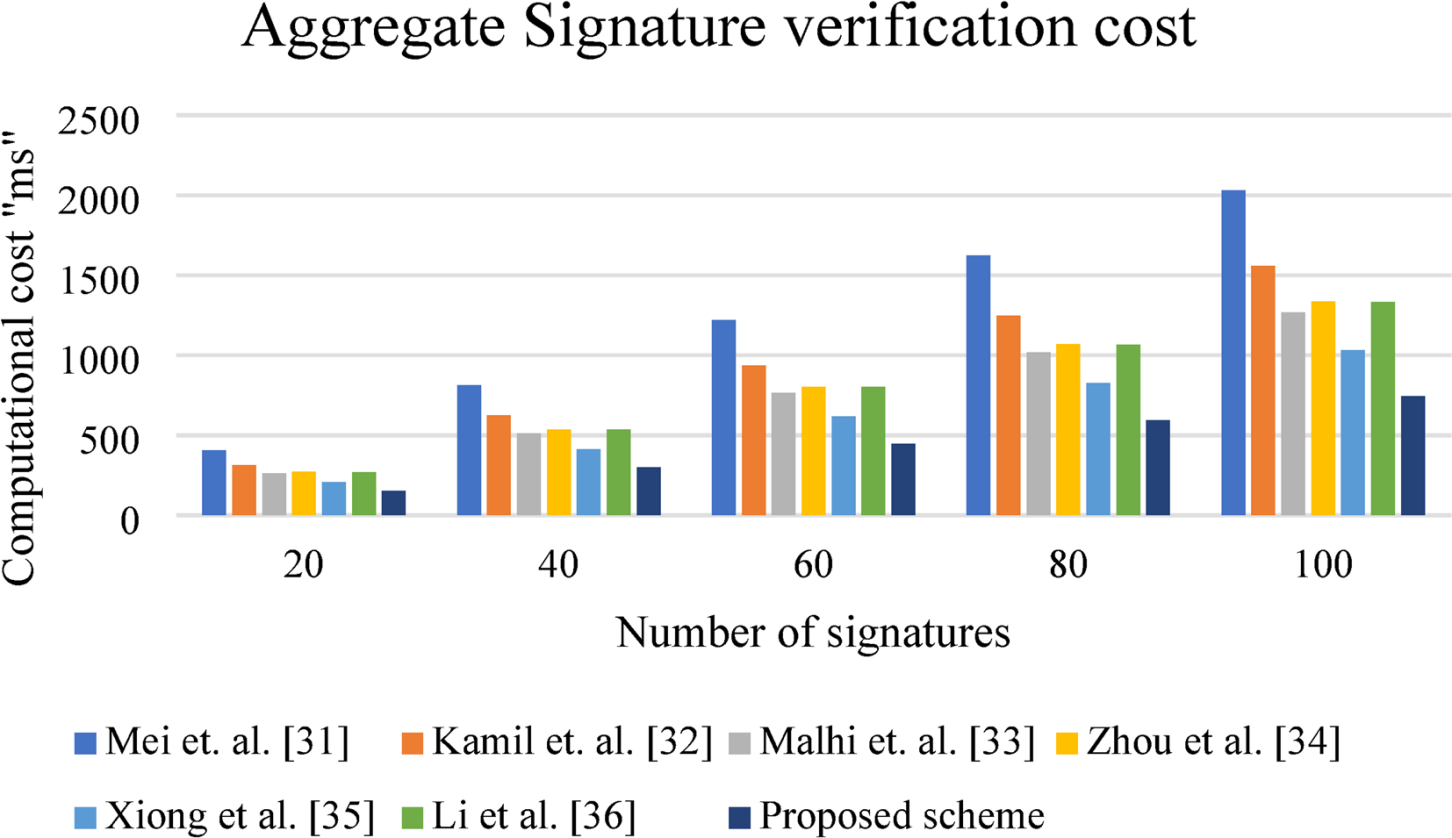
Computational overhead for the increase in the number of signatures.

The time required for performing the cryptographic operation is termed as computational cost/ overhead. In this work, the computational cost is computed for signature generation, signature verification and aggregate signature validation. The execution is performed using Cygwin platform using PBC library^30^. The execution time required for computing one point multiplication ($\:{T}_{pm}$), bilinear pairing operation ($\:{T}_{bp}$), point addition ($\:{T}_{a}$), hashing operation ($\:{T}_{h}$) are computed as $\:2.247\:milliseconds\:\left(ms\right),\:3.21ms,\:0.029ms$ and $\:2.91ms$. The above required computational cost is computed using the system having the following configuration of processor 13th Gen Intel(R) Core(TM) i5-1335U with 1.30 GHz, 8 GB RAM. In this work, the signature is computed as $\:{\sigma\:}_{j}=({X}_{j},{Z}_{j})$, where the computation involves one point multiplication, two hashing operation and two-point addition operation. Similarly, for the signature verification, the vehicle user checks the condition $\:{Z}_{j}{puk}_{TA}={Y}_{j}{L}_{j}+{X}_{j}$, by computing $\:{Y}_{j}^{*}=H(LBI,{DID}_{{R}_{j}},{X}_{j},\:{tt}_{j})$. Thus, during signature validation, one point multiplication and one hashing operation are required. Finally, during the aggregate signature validation, $\:n$ point multiplication and one hashing operation is required for verification of $\:{\Omega\:}$. Further, $\:n$ point multiplication is required for validation of $\:\sum\:_{j=1}^{n}{Z}_{j}{puk}_{TA}$. The proposed scheme is compared with relevant works like Mei et al.^31^, Kamil et al.^32^, Malhi et al.^33^, Zhou et al.^34^, Xiong et al.^35^ and Li et al.^36^ schemes. Table 2 shows the computational cost for single signature generation, verification, and aggregate signature validation for the proposed scheme with the relevant schemes. Figure 4 shows the schematic view of signature generation and verification cost for different pertinent works. This figure clearly indicates that the time required for signature generation and verification for the suggested protocol is comparatively lower than for pertinent works. Figure 5 portrays the pictorial representation for aggregate signature verification. This figure clearly signifies that as the number of signatures increases, the suggested scheme outperforms the relevant schemes. Figure 4 shows the signature generation and verification times (in milliseconds) for different schemes. As observed, our proposed scheme achieves a signature generation time of 8.096 ms and a verification time of 5.157 ms, outperforming all compared methods. For instance, compared to Xiong et al. (7.462 ms generation, 10.314 ms verification), our scheme significantly reduces the verification time by approximately 50%. Figure 5 presents the aggregate signature verification time for various schemes as the number of vehicles increases from 20 to 100. The results show that our proposed scheme consistently achieves the lowest computational overhead across all scenarios. At 20 vehicles, the proposed scheme achieves a verification time of 150.99 ms, significantly lower than Mei et al. (405.98 ms) and Kamil et al. (313.407 ms). At 100 vehicles, the proposed scheme still maintains 743.31 ms, while the others exceed 1000 ms. On average, the proposed scheme improves computational efficiency by approximately 20–60% compared to other existing methods.

**Table 2:** Computational overhead for signature generation, verification and aggregate validation.

S.no.	Schemes	Sign. generation	Sign. verification	Aggregate validation
1	Mei et al.^31^	$\:4{T}_{pm}+3{T}_{h}+{2T}_{a}$	$\:4{T}_{bp}+{2T}_{pm}+{T}_{h}$	$\:4n{T}_{p}+2n{T}_{pm}+{nT}_{h}+(2n-2){T}_{a}$
2	Kamil et al.^32^	$\:3{T}_{pm}+{3T}_{h}+{2T}_{a}$	$\:4{T}_{pm}+{3T}_{a}+{3T}_{h}$	$\:(3n+1){T}_{pm}+{3nT}_{a}+3n{T}_{h}$
3	Malhi et al.^33^	$\:4{T}_{pm}+{2T}_{h}+{2T}_{a}$	$\:3{T}_{bp}+3{T}_{pm}+{T}_{a}+{2T}_{h}$	$\:3{T}_{bp}+3n{T}_{pm}+{nT}_{a}+{2nT}_{h}$
4	Zhou et al.^34^	$\:3{T}_{pm}+2{T}_{h}$	$\:4{T}_{pm}+3{T}_{a}+3{T}_{h}$	$\:(2n+2){T}_{pm}+3n{T}_{a}+3n{T}_{h}$
5	Xiong et al.^35^	$\:2{T}_{pm}+{T}_{h}+2{T}_{a}$	$\:2{T}_{pm}+2{T}_{h}$	$\:2n{T}_{pm}+2n{T}_{h}$
6	Li et al.^36^	$\:{T}_{pm}+{2T}_{h}+{T}_{a}$	$\:{3T}_{pm}+{3T}_{h}+3{T}_{a}$	$\:(2n+1){T}_{pm}+3n{T}_{h}+3n{T}_{a}$
7	Proposed work	$\:{T}_{pm}+{2T}_{h}+{T}_{a}$	$\:{T}_{pm}+{T}_{h}$	$\:2n{T}_{pm}+(n+1){T}_{h}$

### Communication overhead

**Fig. 6:**
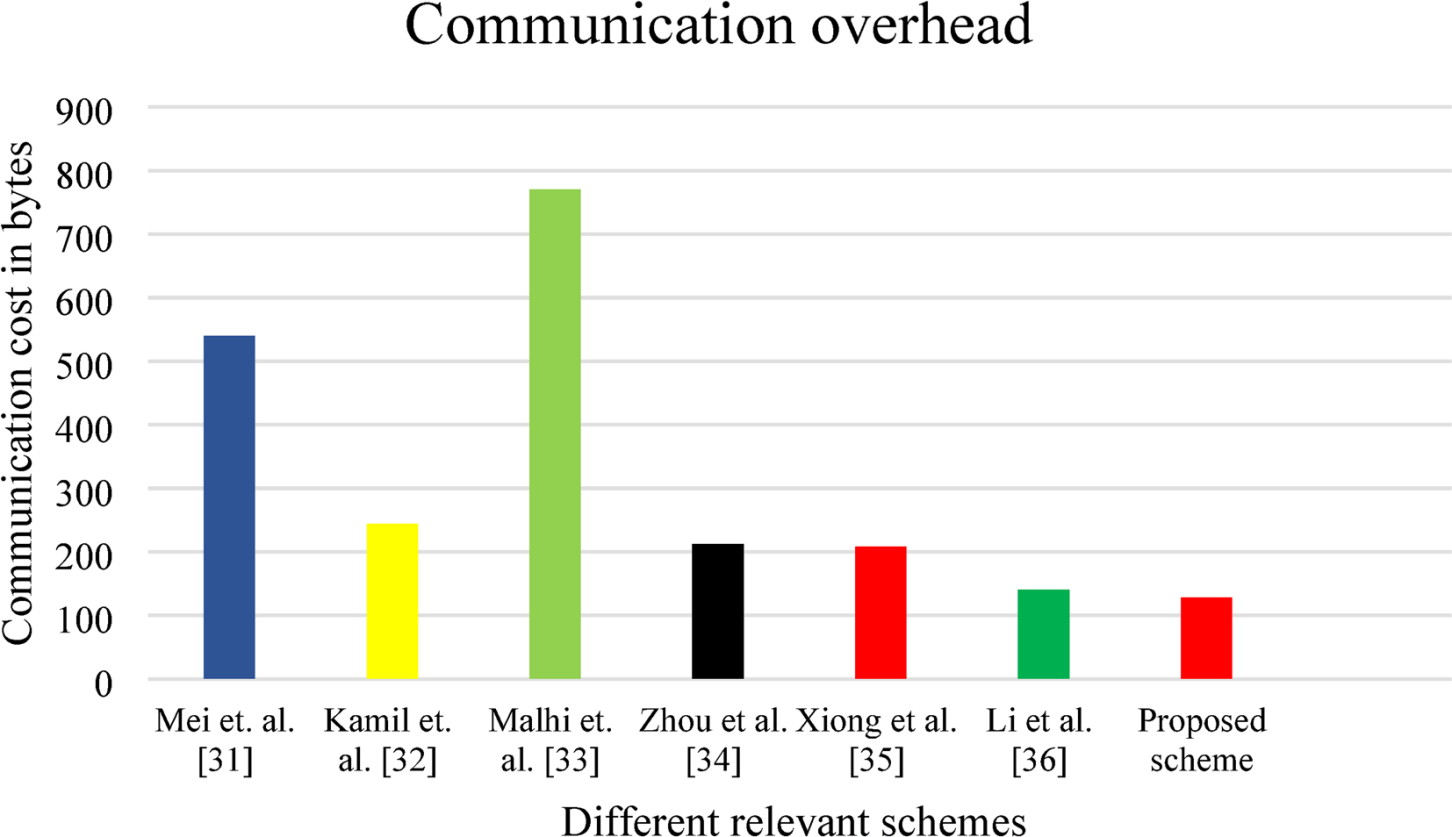
Different relevant works communication cost in bytes.

The number of bytes required during LBI transmission is referred as communication cost. In this work, $\:{RSU}_{j}$ shares $\:(LBI,\:{tt}_{j},{\sigma\:}_{j},{L}_{j})$ to the $\:{V}_{i}$. The elements belong to the $\:G$ and $\:{Z}_{q}^{*}\:$are have the element size of 40 and 20 bytes. The output of the hash function and timestamp are 20 and 8 bytes respectively. Table 3 shows the communication cost of the suggested protocol with Mei et al.^31^, Kamil et al.^32^, Malhi et al.^33^, Zhou et al.^34^, Xiong et al.^35^ and Li et al.^36^ schemes. Figure 6 shows the schematic view with respect to relevant work. Form the figure, it is clear that the suggested work requires minimum number of bytes for transferring the LBI to the $\:{V}_{i}$. As shown in Table 3, our proposed scheme achieves a communication overhead of 208 bytes, which is among the lowest compared to existing approaches. Specifically: The proposed scheme reduces communication overhead by 61.48% compared to Mei et al. (540 bytes), and by 73% compared to Malhi et al. (770 bytes). The low communication overhead stems from the use of aggregate signatures and optimized certificateless mechanisms, which avoid transmitting multiple certificates and redundant authentication materials.

**Table 3:** Different relevant works communication overhead.

S.no.	Schemes	Communication cost (bytes)
1	Mei et al.^31^	540
2	Kamil et al.^32^	244
3	Malhi et al.^33^	770
4	Zhou et al.^34^	212
5	Xiong et al.^35^	208
6	Li et al.^36^	140
7	Proposed work	128

The security attributes of the state-of-the-art schemes is shown in Table 4. The table clearly indicates the secure and robustness of the proposed protocol against different schemes.

**Table 4:** Comparison of security attributes.

Security	^31^	^32^	^33^	^34^	^35^	^36^	Proposed
Anonymity	$\:\text{{\rm\:Y}}$	$\:\times\:$	$\:\text{{\rm\:Y}}$	$\:\text{{\rm\:Y}}$	$\:\text{{\rm\:Y}}$	$\:\text{{\rm\:Y}}$	$\:\text{{\rm\:Y}}$
Unlinkability	$\:\text{{\rm\:Y}}$	$\:\text{{\rm\:Y}}$	$\:\text{{\rm\:Y}}$	$\:\text{{\rm\:Y}}$	$\:\times\:$	$\:\text{{\rm\:Y}}$	$\:\text{{\rm\:Y}}$
Traceability	$\:\text{{\rm\:Y}}$	$\:\times\:$	$\:\text{{\rm\:Y}}$	$\:\text{{\rm\:Y}}$	$\:\text{{\rm\:Y}}$	$\:\text{{\rm\:Y}}$	$\:\text{{\rm\:Y}}$
Replay attack	$\:\text{{\rm\:Y}}$	$\:\text{{\rm\:Y}}$	$\:\text{{\rm\:Y}}$	$\:\text{{\rm\:Y}}$	$\:\text{{\rm\:Y}}$	$\:\text{{\rm\:Y}}$	$\:\text{{\rm\:Y}}$
Modification attack	$\:\text{{\rm\:Y}}$	$\:\text{{\rm\:Y}}$	$\:\text{{\rm\:Y}}$	$\:\text{{\rm\:Y}}$	$\:\text{{\rm\:Y}}$	$\:\text{{\rm\:Y}}$	$\:\text{{\rm\:Y}}$
Sybil attack	$\:\times\:$	$\:\times\:$	$\:\times\:$	$\:\times\:$	$\:\times\:$	$\:\times\:$	$\:\text{{\rm\:Y}}$
MITM attack	$\:\times\:$	$\:\text{{\rm\:Y}}$	$\:\times\:$	$\:\text{{\rm\:Y}}$	$\:\text{{\rm\:Y}}$	$\:\text{{\rm\:Y}}$	$\:\text{{\rm\:Y}}$
DOS	$\:\times\:$	$\:\times\:$	$\:\times\:$	$\:\text{{\rm\:Y}}$	$\:\text{{\rm\:Y}}$	$\:\times\:$	$\:\text{{\rm\:Y}}$
Mutual authentication	$\:\times\:$	$\:\times\:$	$\:\text{{\rm\:Y}}$	$\:\text{{\rm\:Y}}$	$\:\times\:$	$\:\times\:$	$\:\text{{\rm\:Y}}$
Scalability	$\:\times\:$	$\:\times\:$	$\:\times\:$	$\:\times\:$	$\:\times\:$	$\:\times\:$	$\:\text{{\rm\:Y}}$

$$\:\text{{\rm\:Y}}-Yes\times\:-No$$

### Practical implementation and parametric outcomes

**Fig. 7:**
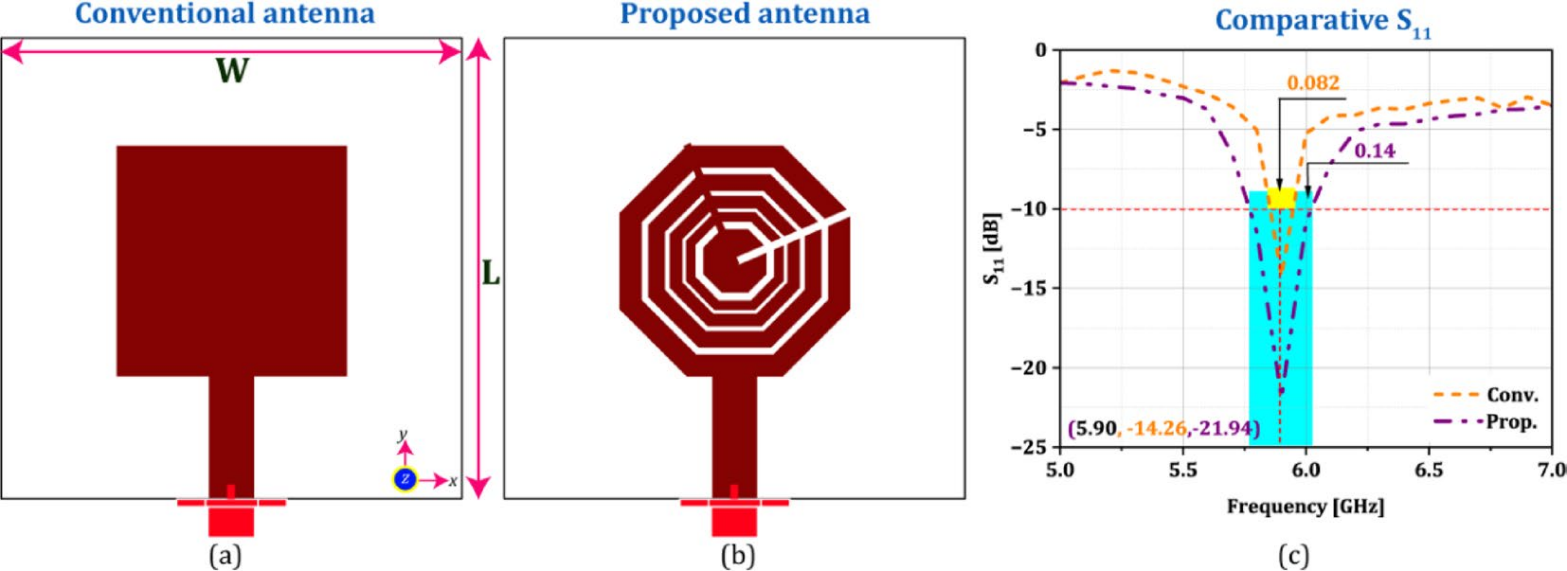
(**a**) Conventional and (**b**) proposed antenna module (**c**) comparative S_11_.

The designed antenna is placed on the OBU of the $\:{V}_{i}$ and $\:{RSU}_{j}$ for efficient communication of LBI. To practically validate the efficiency of the designed antenna, it is simulated using CST Microwave Studio software, v2019^37^. The different characteristics of the proposed newly designed miniature antenna with respect the standard parameter is mentioned in the Table 4 as follows. Additionally, we’ve also simulated a conventional square antenna (with identical aperture area) and compared the results with our proposed one. The structural comparison as well as the comparative performances has been carried out in Fig. 7; Table 4, respectively in the revised manuscript. It can be clearly observed that the proposed Golden Ratio-based antenna offers better parametric outcomes compared to the conventional one. This table also highlighted that the proposed printed antenna module is suitable candidature for the desired application especially VANET.

**Table 4:** Comprehensive parametric outcomes of conventional and proposed antenna module.

Parameters	Recommended value	Conventional antenna	Proposed antenna
Antenna resonance [GHz]	5.90	5.90	5.90
S_11_[dB]	<-10	-14.26	-21.94
Impedance bandwidth [GHz]	0.075	0.082	0.14
Antenna gain [dBi]	3	3.67	7.42
Cross polarization[dB]	<-20	E=-21.23, H=-22.46	E=-33.47, H=-37.28
Back-lobe [dB]	<-15	-21	-30
Radiation efficiency [%]	> 80	83	92

**Fig. 8:**
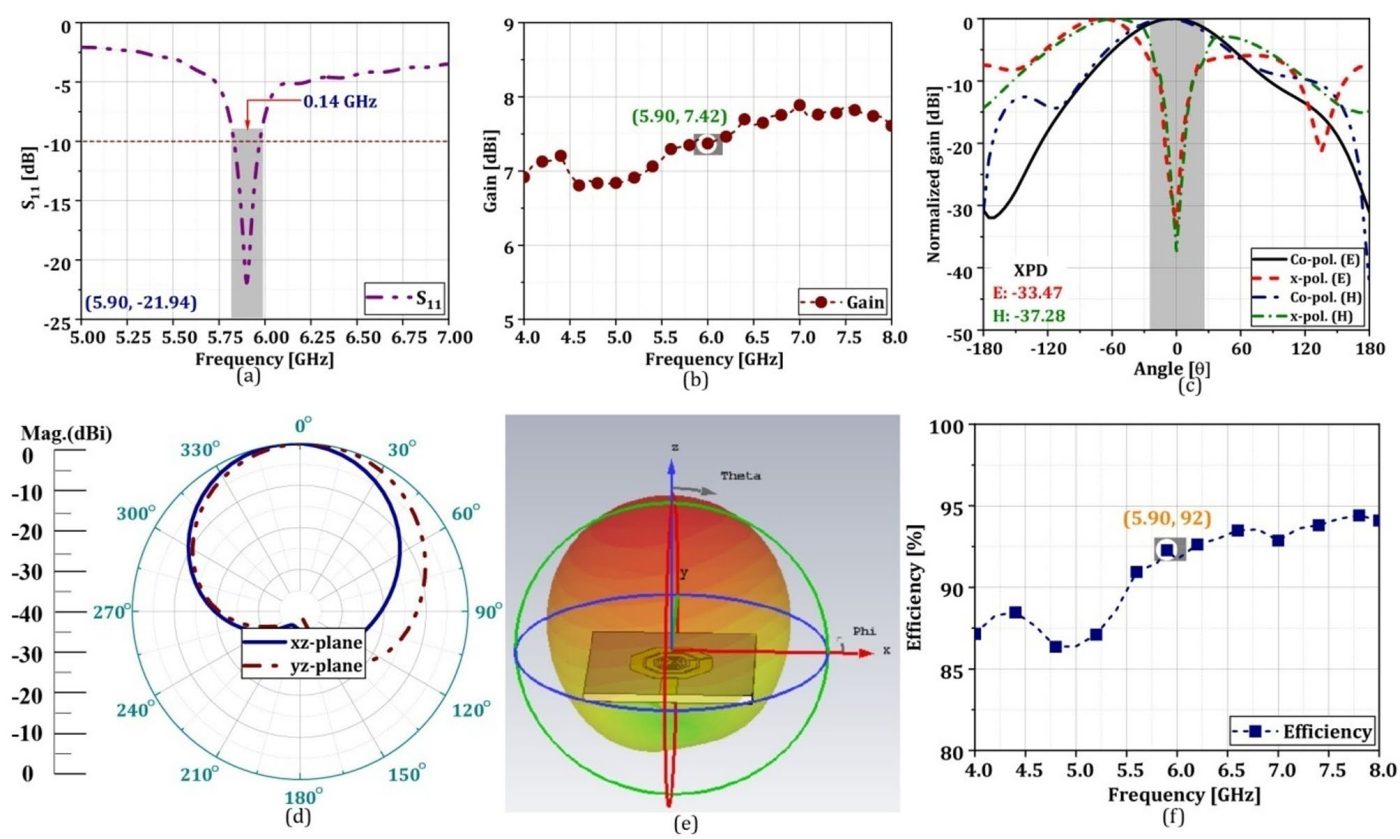
(**a**) S_11_ (**b**) gain (**c**) E & H-field, (**d**) xz-yz (**e**) 3D-radiation patterns and (**f**) efficiency of antenna. The suggested antenna has successfully produced a symmetric radiation pattern, as seen in Fig. 8(e). Further, the 3D radiation pattern is immediately retrieved from the CST microwave studio for further clarification. It can be clearly indicated that the proposed antenna offers a symmetric radiation pattern in the desired direction, which is widely acceptable for such types of applications. The antenna also offers 92% radiation efficiency at the designated operation frequency. The radiation efficiency of the antenna is graphically presented in Fig. 8(f). It can be clearly observed that the antenna gives 92% radiation efficiency at a particular frequency of 5.90 GHz. The proposed system with higher radiation capabilities provides many advantages, such as extended signal range, better signal quality, increased energy efficiency, decreased heat generation, adherence to regulatory standards, compact design, and the possibility for cost savings.

The return-loss (S11) characteristics are shown in Fig. 8(a). The figure clearly shows that the antenna’s resonance occurs at a frequency of 5.90 GHz, exhibiting a return loss of 21.94 dB. This means that at least 80% of the power is transmitted out of the antenna, and only 20% is reflected back. The attained return loss of 21.94 dB will ensure that the antenna efficiently transmits the power in the desired direction and that there is very minimal signal loss. For a miniature antenna operating in DSRC, a minimum impedance bandwidth of at least 75 MHz is generally recommended. DSRC operates in the 5.90 GHz band, encompassing a range of frequencies from 5.850 GHz to 5.925 GHz. An impedance bandwidth of 75 MHz ensures that the antenna can effectively operate within this entire frequency range. The designed antenna achieves an impedance bandwidth of 0.14 GHz, which is sufficient to include the DSRC band. In DSRC, as it is a short-wave communication system, an antenna with a 3 dBi gain is generally recommended for the resonating frequency of 5.90 GHz. The proposed designed antenna offers 7.42 dBi gain at the specific frequency, which is depicted in Fig. 8(b). This ensures that the designed antenna has a higher gain, which can effectively transmit and receive signals within the short range required for DSRC applications, which primarily involve V2V and V2I communications. Figure 8(c) visually illustrates the graphical representation of the attributes of the E and H-fields. The figure clearly indicates that the designed antenna exhibits a cross-polarization discrimination of E = -33.47 and H = -37.28, which are less than − 20 dB for the respective electric and magnetic fields. As a result, the use of VANET applications inside the DSRC band (802.11p) is widely acknowledged. Figure 8(d) illustrates the radiation patterns of the antenna in the xz-yz plane in the required direction, with a phase angle of 0° and 90°. The antenna exhibits − 30 dB back-lobe radiation, which is less than − 15 dB (typical value), which indicates there will be a minimum back wave’s propagation with maximum directivity.

## Conclusion

An efficient mutual certificateless anonymous authentication is suggested in this work, where conditional privacy and integrity are preserved. A novel miniature printed radiator in the form of an octagonal ring, based on the ‘Golden ratio’ principle, is proposed for use in VANETs. Security against various well-known attacks is explained in the security investigation section. A novel aggregate signature verification scheme with minimum computational and communication overhead is proposed when compared to relevant schemes. By solving existing constraints in computing and communication performance, the suggested scheme directly improves future Intelligent Transportation Systems safety, efficiency, and deployment readiness. Finally, the designed antenna has a reduced size of 19.76%. As a result, the single radiator is capable to cover the entire DSRC band. It also exhibits satisfactory gain, very low cross-polarization, and good radiation efficiency compared to conventional printed antenna operating at desired frequency of 5.90 GHz. This antenna module is also capable to offer an effective frequency transmission in the DSRC band, especially for Vehicular Ad-hoc Networks (VANETs).

## Data Availability

The datasets used and/or analysed during the current study available from the corresponding author on reasonable request.
